# Comprehensive Analyses of circRNA Expression Profiles and Function Prediction in Chicken Cecums After *Eimeria tenella* Infection

**DOI:** 10.3389/fcimb.2021.628667

**Published:** 2021-03-10

**Authors:** Hailiang Yu, Changhao Mi, Qi Wang, Wenbin Zou, Guojun Dai, Tao Zhang, Genxi Zhang, Kaizhou Xie, Jinyu Wang, Huiqiang Shi

**Affiliations:** ^1^ College of Animal Science and Technology, Yangzhou University, Yangzhou, China; ^2^ Technical Research Department, Jiangsu Jinghai Poultry Group Co. Ltd., Haimen, China

**Keywords:** chicken, circRNAs, *E. tenella*, cecum, expression profiles

## Abstract

Coccidiosis is an important intestinal parasitic disease that causes great economic losses to the global poultry production industry. Circular RNAs (circRNAs) are long non-coding RNAs that play important roles in various infectious diseases and inflammatory responses. However, the expression profiles and functions of circRNAs during *Eimeria tenella* (*E. tenella*) infection remain unclear. In this study, high-throughput sequencing was carried out to detect circRNAs in chicken cecal tissues from the control (JC), resistant (JR), and susceptible (JS) groups on day 4.5 postinfection (pi), respectively. A total of 104 circRNAs were differentially expressed, including 47 circRNAs between the JS and JC groups, 38 between the JR and JS groups, and 19 between the JR and JC groups. Functional analyses indicated that these differentially expressed circRNAs were involved in pathways related to *E. tenella* infection; the adaptive immune response was enriched in the JS *vs* JC group, the NF-kappa B signaling and natural killer cell-mediated cytotoxicity pathways were enriched in the JS *vs* JC and JR *vs* JC groups, while the B cell receptor signaling pathway was enriched in only the JR *vs* JC group. Moreover, the coexpression network of differentially expressed circRNAs and mRNAs suggested that circRNA2202 and circRNA0759 associated with *DTX1* in the JS *vs* JC group, circRNA4338 associated with *VPREB3* and *CXCL13L3* in the JR *vs* JC group, and circRNA2612 associated with *IL8L1* and *F2RL2* in the JR *vs* JS group were involved in the immune response upon *E. tenella* infection. In conclusion, our results provide valuable information on the circRNAs involved in the progression of chicken *E. tenella* infection and advance our understanding of the circRNA regulatory mechanisms of host resistance and susceptibility to *E. tenella* infection in chickens.

## Introduction

Avian coccidiosis is an intestinal parasitic disease caused by *Eimeria* protozoa and hinders the development of the global poultry industry (Shirley and Lillehoj, 2012). The global poultry industry has annual economic losses of up to $3 billion due to coccidiosis. Seriously, the subclinical economic losses caused by infected chickens are even more severe, including the impact on weight gain and egg production (Sharman et al., 2010; Blake and Tomley, 2014). To date, there are seven species of *Eimeria* that have been recognized worldwide, and each genus of coccidia is parasitic in a specific region. *E. tenella* parasitizes the chicken cecum, mainly causing bleeding of the cecum epithelium and bloody stools (Yu et al., 2019). In addition, due to the high tolerance and survival rate of coccidia oocysts in the environment, *E. tenella* is common in the poultry industry. At present, the prevention strategies for coccidiosis are mainly divided into three types: anticoccidial drugs, vaccines, and strict feeding hygiene management (Swaggerty et al., 2015). Drug resistance issues and violations of antibiotic bans have attracted much attention (Xie et al., 2020). In this regard, selecting breeds and lines with natural resistance to coccidiosis based on the genetic variability of chickens and genes related to resistance is an effective, long-term prevention strategy (Kim et al., 2009). Genetically distinct lines of broilers have shown differences in resistance or susceptibility to *Salmonella enteritidis* (Swaggerty et al., 2005), *Campylobacter jejuni* (Li et al., 2008), and *E. tenella* (Swaggerty et al., 2011) infections. Broiler breeders with an efficient innate immune response are more resistant to *E. tenella* (Swaggerty et al., 2011), and a novel selected mean based on a higher phenotype of some pro-inflammatory mediators was formed to produce broilers that are naturally more resistant to *E. tenella* (Swaggerty et al., 2015).

Circular RNAs (circRNAs) are a special type of endogenous non-coding RNA that are widespread in animals and plants (Li et al., 2019). Compared to linear RNA, circRNA is formed through the back-splicing of covalently bound 3′-and 5′-ends. Therefore, they are more highly conserved and stable (Jeck et al., 2013). Previous studies have shown that circRNAs can play a regulatory role as miRNA sponges and can also act as posttranscriptional regulators and templates for translating proteins to perform biological functions (Hansen et al., 2013; Jeck et al., 2013; Memczak et al., 2013). Increasing evidence has shown that circRNAs play an important regulatory role in pathological processes such as *Alzheimer’s* disease, cancer, and viral infections (Li et al., 2015; Li et al., 2017; Cervera-Carles et al., 2020). However, the expression and regulatory mechanisms of circRNAs during *E. tenella infection* are unclear. In this study, we first investigated the expression profile and function of circRNAs in cecal tissues of broilers infected with *E. tenella* in different groups (the control, resistant, and susceptible groups) to understand the complex mechanisms of resistance and susceptibility to *E. tenella* mediated by circRNAs.

## Materials and Methods

### Samples and Challenge

**Figure 1:**
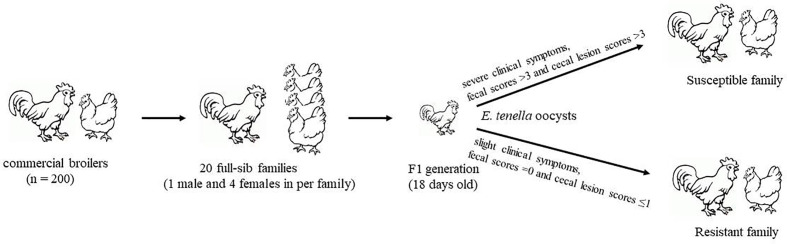
Workflow of sample selection.

A total of 20 full-sib family broilers were selected from 200 Jinghai Yellow Chickens at Jinghai Yellow Chicken Resource Farm in Jiangsu Province, China. One male and four female parents in each family were artificially fertilized to produce the F1 generation (the number of F1 generations of each family was not less than 10). The offspring of each family were raised separately in oocyst-free cages and fed antibiotic-free feed and water. At 18 days old, F1 individuals from each family were randomly divided into two groups: the treatment group and the control group. Each chicken in the treatment group was orally infected with the same dose of sporulated *E. tenella* oocysts (3.5 × 10^4^ oocysts/bird). In this study, the clinical symptoms (emaciation, drooping wings, and dying death), fecal scores (Morehouse and Baron, 1970), and cecal lesion scores (Johnson and Reid, 1970) were used together to discriminate susceptible (JS, severe clinical symptoms, fecal scores >3, and cecal lesion scores >3) from resistant (JR, slight clinical symptoms, fecal scores = 0, and cecal lesion scores ≤1) birds. Thus, the most resistant and most susceptible families were selected for subsequent sequencing (**Figure 1**). Each chicken in the control group of the resistant family received the same amount of normal saline (JC).

Parasite oocysts were collected from the Department of Parasitology, College of Veterinary Medicine, Yangzhou University (Jiao et al., 2018). On the 4.5th day postinfection [the point time to identify the resistance and susceptibility chickens according to whether cecum bleeding or not (Rose and Hesketh, 1976)], the cecal tissues of the three broilers in the JS, JR, and JC groups were collected, snap-frozen, and preserved at −80°C for later use. All animal protocols were approved by the Animal Welfare Committee of Yangzhou University, and all efforts were made to minimize the suffering of the chickens.

### Library Construction and High‐Throughput Sequencing Analysis

Total RNA from cecal samples in each group was extracted using TRIzol reagent (Invitrogen, Carlsbad, CA, USA) according to the manufacturer’s instructions. The purity of RNAs was checked using a NanoPhotometer^®^ spectrophotometer (IMPLEN, CA, USA). The concentration and integrity of RNAs were determined using the Qubit^®^ RNA Assay Kit in Qubit^®^ 2.0 Flurometer (Life Technologies, CA, USA) and Bioanalyzer 2100 system (Agilent Technologies, CA, USA), respectively. The quality of the RNA samples met the experimental requirements (RIN> = 7 28S/18S> = 0.7) and were used for RNA sequencing.

Ribosomal RNA was removed using the Ribo-Zero Gold rRNA Removal Kit (Illumina), and the remaining RNA was fragmented by a fragmentation reagent. Then, the libraries were constructed using TruSeq Stranded Total RNA with Ribo-Zero Gold (Illumina, Cat. no. RS-122-2301) according to the manufacturer’s instructions. Whole transcriptome sequencing, including circRNA sequencing and mRNA sequencing, was carried out on an Illumina HiSeq 2500 (OE Biotech, Shanghai, China), and the reading length was 2 × 150 BP (pe150).

### Differentially Expressed circRNA and mRNA Analysis

The clean reads were aligned to a reference genome using HISAT2 (Kim et al., 2015). Then, CIRI (v2.0.3) was employed to detect and identify the circRNAs (Gao et al., 2015). The expression of circRNAs was calculated using spliced reads per million mapped reads (RPM). For mRNAs, Cufflinks (Trapnell et al., 2010) was used to calculate the fragments per kilobase of transcript per million mapped reads (FPKM) value (Roberts et al., 2011) of expression of each gene, and the read counts of each gene were obtained by HTSeq-count (Anders et al., 2015). The DESeq (2012) R package was also used to perform differential expression analysis. Finally, the differentially expressed circRNAs and mRNAs were screened based on fold change (FC) > 2, and the *P-value* was <0.05.

### CircRNA Functional Analysis

Gene Ontology (GO) analysis was performed to determine the basic functions of the host genes of differentially expressed circRNAs, including three classes: biological process (BP), cellular component (CC), and molecular function (MF). The Kyoto Encyclopedia of Genes and Genomes (KEGG) was used to analyze the functional pathways of the host genes of the circRNAs. GO terms and KEGG pathways were considered to be significantly enriched based on a *P-value <*0.05.

### Construction of the circRNA-mRNA Coexpression Network

We used the Pearson correlation coefficient (*PCC*) to calculate the correlation between differentially expressed circRNAs and differentially expressed mRNAs and constructed a coexpression network. CircRNA-mRNA interaction pairs were extracted with the value of *PCC* ≥0.8 and *P-value <*0.05, and the top 100 coexpression of circRNA-mRNA network map was then constructed using the R network package.

### Real-Time PCR

Total RNA was extracted from original cecal tissues and used to synthesize cDNA using an RT-PCR Kit (TaKaRa Biotech, Dalian). Seven circRNAs were selected to verify the reliability of RNA-Seq, including six circRNAs, and β-actin was used as an internal reference gene. Primer sequences (**Table 1**) were synthesized by Sangon Biotech Co., Ltd. (Shanghai, China). Then, qRT-PCR was performed using SYBR Green (TaKaRa Biotech, Dalian). The reactions were carried out as follows: initial denaturation at 94°C for 30 s, followed by 40 cycles of denaturation for 5 s at 95°C and annealing for 34 s at 60°C. Data at multiple points were collected for dissolution curve analysis, and the procedure was as follows: 95°C for 15 s, 60°C for 1 min, 95°C for 15 s, and 60°C for 15 s. Each sample was analyzed in triplicate. The qRT-PCR results were analyzed using the 2^−ΔΔCt^ method (Livak and Schmittgen, 2001).

**Table 1:** Primer sequences for differentially expressed circRNAs.

circRNAs	Primer sequences (5’-3’)		Length/bp
β-actin		F: CCTGAACCTCTCATTGCCA R: GAGAAATTGTGCGTGACATCA	152
circRNA_4375|Chr31:5251588_5267684_-		F: CTTAGAAACAACGAGGGACGA R: TCAGGACTCACAGTGATGGGA	183
circRNA_6530|ChrZ:38536561_38537174_+		F: AGTCACACCTCCCGATCAAGG R: GGTGCGGAGTGAGAGGTACA	121
circRNA_6300|ChrW:4831150_4838596_+		F: TGCAGACCAGATACGGGGTA R: CCCAAGCACTGGAAGGGCTA	136
circRNA_0759|Chr1:130466378_130493459_-		F: GGACATTGCTATGTTTGCAGG R: ACATCACCACCTCTCGAGTTC	162
circRNA_4340|Chr31:2362269_2405166_-		F: CCATAAAAGCACCGGCTCTC R: CATATTCCAACCCCTTGCCG	250
circRNA_1909|Chr15:8218348_8230982_-		F: GGGTATAAAAGGGCATCGAG R: AGCCATAACCATAGCCACTG	227

bp, base pair; circRNA, circular RNA; F, forward; R, reverse.

## Results

### Identification of circRNAs in Cecal Tissues by RNA-Seq

**Figure 2:**
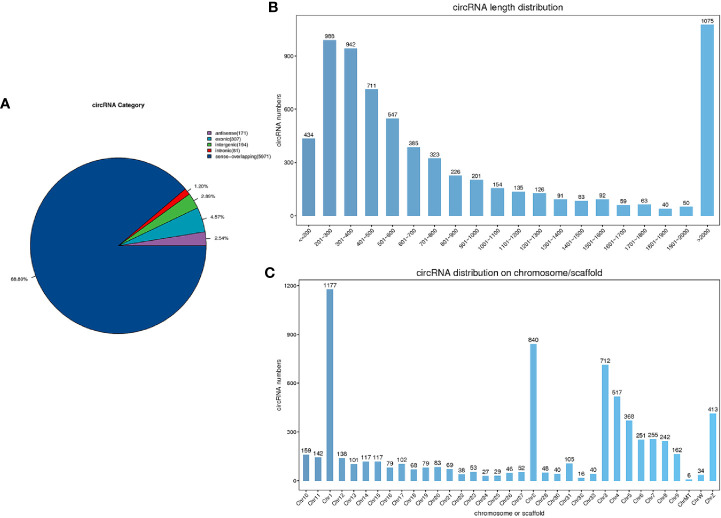
Characteristics of circRNAs in chicken cecal tissues during *E. tenella* infection. **(A)** circRNA category. **(B)** The length distribution of circRNAs. **(C)** Chromosomal distribution of circRNAs.

We obtained 1000.44 M raw reads from nine libraries by Illumina sequencing. After removing adapter reads and low-quality reads, a total of 967.44 M clean reads were used for the identification of circRNAs. The accuracy of base recognition (Q30) in each sample was greater than 95% (**Table 2**). A total of 6,725 circRNAs were identified in all nine cecal samples of broilers (JR *vs* JC, 5,439; JR *vs* JS, 5,752; JS *vs* JC, 5,352). The majority of them were sense-overlapping circRNAs (88.80%), while a small proportion (1.20%) of circRNAs contained intronic sequences (**Figure 2A**). In addition, the lengths of these circRNAs were mostly concentrated at 200–2,000 nt (**Figure 2B**), which was consistent with the reported length distribution of circRNAs. Most of the circRNAs were distributed on chromosome 1, followed by distribution on chromosomes 2 and 3 (**Figure 2C**).

**Table 2:** Sequencing data and quality parameters.

Sample	Raw reads	Clean reads	Multiple mapped (%)	Uniquely mapped (%)	Q30 (%)	GC (%)
JC1	107.54 M	104.92 M	6299668 (6.00)	93010583 (88.65)	96.13	47.73
JC2	109.24 M	105.23 M	13308196 (12.65)	84687626 (80.48)	95.89	53.21
JC3	111.36 M	107.18 M	8417074 (7.85)	93783447 (87.50)	95.86	48.32
JR1	110.64 M	107.46 M	7548912 (7.02)	94792432 (88.21)	95.81	48.05
JR1	102.58 M	100.09 M	6540337 (6.53)	88766317 (88.69)	96.21	47.62
JR1	117.57 M	114.35 M	9002737 (7.87)	99731881 (87.22)	96.01	47.96
JS1	115.24 M	110.56 M	8323574 (7.53)	97330051 (88.03)	95.55	47.73
JS2	110.87 M	106.66 M	7483362 (7.02)	93909225 (88.04)	95.77	47.61
JS3	115.40 M	110.99 M	9413810 (8.48)	96283860 (86.75)	95.77	48.21

M, the size of the data volume; Q30, the percentage of bases with a Qphred value greater than 30 in Raw base to the total base; GC, the percentage of the total number of G and C in CleanBase to the total number of bases.

### Differentially Expressed circRNAs in Chicken Cecal Tissues During *E. tenella* Infection

**Figure 3:**
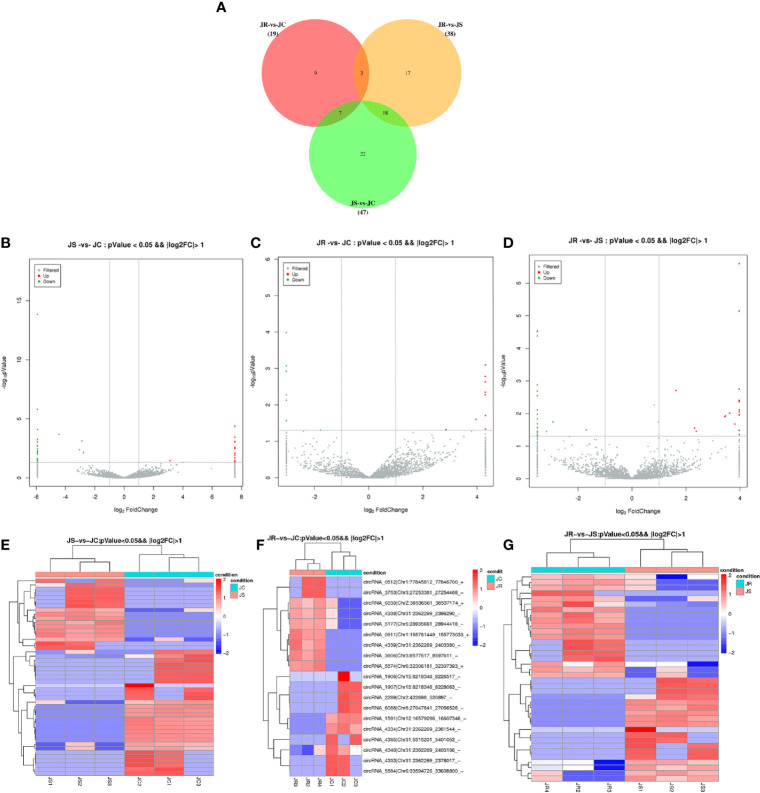
Expression profiles of differentially expressed circRNAs in chicken cecal tissues of different groups during *E. tenella* infection. **(A)** Venn diagram of differentially expressed circRNAs. **(B–D)** Volcano plots of differentially expressed circRNAs in the JS *vs* JC group **(B)**, JR *vs* JC group **(C)**, and JR *vs* JS group **(D)**. **(E–G)** Hierarchical clustering plots of differentially expressed circRNAs in the JS *vs* JC group **(E)**, JR *vs* JC group **(F)**, and JR *vs* JS group **(G)**.

To further investigate the regulatory role of these circRNAs in cecal tissues challenged with *E. tenella*, we analyzed the differential expression of circRNAs from circRNA expression profiling based on a fold change (FC) > 2 and a *P-value <*0.05.* A* total of 47 (17 upregulated and 30 downregulated), 38 (19 upregulated and 19 downregulated), and 19 (9 upregulated and 10 downregulated) differentially expressed circRNAs were identified in the JS *vs* JC, JR *vs* JS, and JR *vs* JC groups, respectively (**Figures 3B–D**, **Supplementary Table 1**). Three circRNAs showed differential expression in the JR *vs* JS and JR *vs* JC groups, seven circRNAs showed differential expression in the JS *vs* JC and JR *vs* JC groups, and 18 circRNAs showed differential expression in the JR *vs* JS and JS *vs* JC groups (**Figure 3A**). Hierarchical clustering analysis showed that the expression patterns of significantly differentially expressed circRNAs were different between the control group and the treatment group (**Figures 3E–G**).

### GO Enrichment and KEGG Pathway Analyses of Differentially Expressed circRNAs

**Figure 4:**
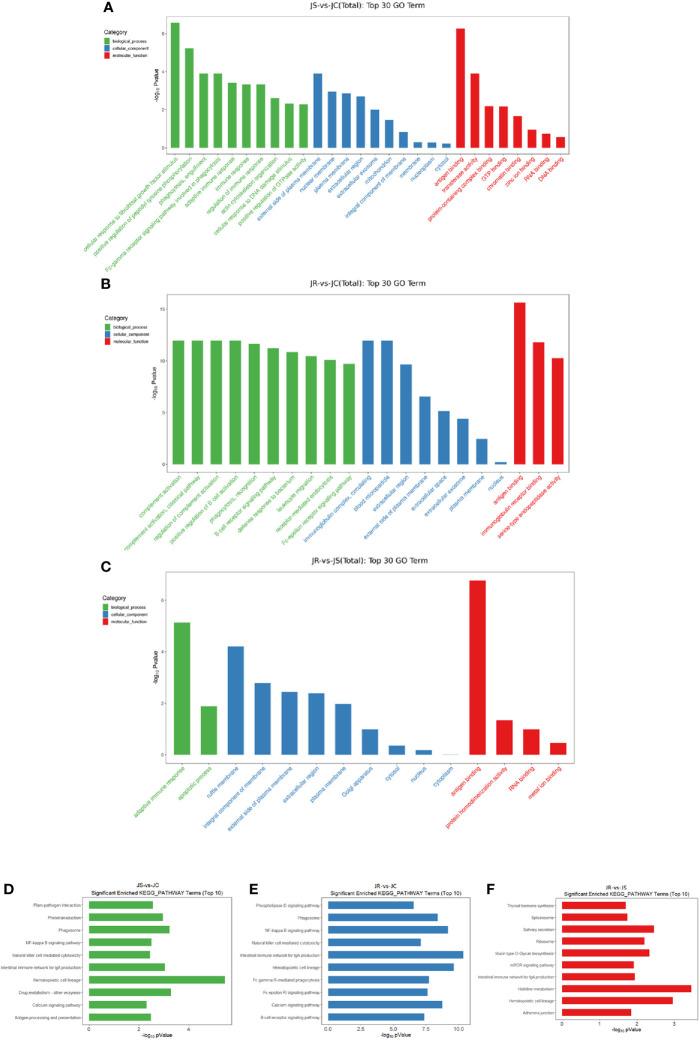
Functional enrichment analysis of differentially expressed circRNAs in chicken cecal tissues of different groups during *E. tenella* infection. **(A–C)** GO term analysis of differentially expressed circRNAs in the JS *vs* JC group **(A)**, JR *vs* JC group **(B)**, and JR *vs* JS group **(C)**. **(D–F)** KEGG pathway analysis of differentially expressed circRNAs in the JS *vs* JC group **(D)**, JR *vs* JC group **(E)**, and JR *vs* JS group **(F)**.

Because most circRNAs are derived from middle exons of protein-coding genes, the processing of circRNAs can affect splicing of their precursor transcripts, resulting in changes in the expression of linear host genes (Li et al., 2018). To explore the functions of differentially expressed circRNAs, host genes of these circRNAs were used for GO and KEGG enrichment analyses. The list of host genes is shown in **Supplementary Table 1**. The top 30 significantly enriched GO terms are shown in **Figure 3** (**Figures 4A–C**, **Supplementary Table 2**). Biological processes, adaptive immune response, immune response, and regulation of immune response were enriched in the JS *vs* JC group. Positive regulation of B cell activation and the B cell receptor signaling pathway were enriched in the JR *vs* JC group. The adaptive immune response and apoptotic process were enriched in the JR *vs* JS group. For the cellular component, the immunoglobulin complex, circulating and blood microparticle were enriched in the JR *vs* JC group. Regarding molecular function, antigen binding was enriched in the JS *vs* JC group, antigen binding and immunoglobulin receptor binding were enriched in the JR *vs* JC group, and antigen binding was enriched in the JR *vs* JS group.

Some signaling pathways that are potentially involved in the regulation of *E. tenella* infection and host resistance were significantly (*P* < 0.05) enriched in the top 10 KEGG pathways in different groups, such as the NF-kappa B signaling pathway and natural killer cell-mediated cytotoxicity in the JS *vs* JC and JR *vs* JC groups, the B cell receptor signaling pathway in the JR *vs* JC group, the intestinal immune network for IgA production and hematopoietic cell lineage in all three groups (**Figures 4D–F**, **Supplementary Table 3**).

### Validation of Differentially Expressed circRNAs

**Figure 5:**
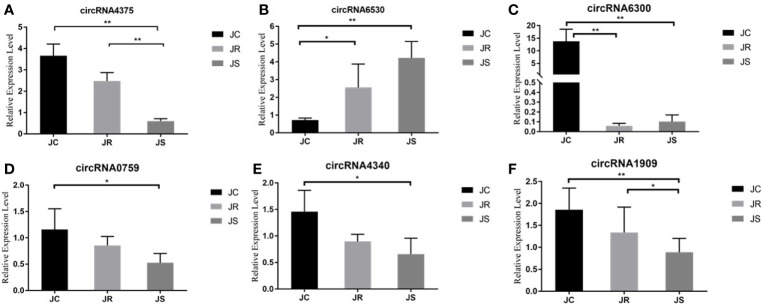
Validation of differentially expressed circRNAs in chicken cecal tissues of different groups during E. tenella infection. Six circRNAs are in the following order: circRNA4375 **(A)**, circRNA6530 **(B)**, circRNA6300 **(C)**, circRNA0759 **(D)**, circRNA4340 **(E)** and circRNA1909 **(F)**. Values are the mean ± SD (n = 3 per group), and qRT-PCR analysis was conducted in triplicate. *P < 0.05, **P < 0.01.

To further verify the reliability of the RNA sequencing results, six circRNAs with high fold changes and a high number of junctions from high-throughput sequencing were randomly selected for qRT-PCR. The qRT-PCR results suggested that circRNA expression using qRT-PCR was consistent with the RNA sequencing results (**Figure 5**).

### The Interaction Network of Differentially Expressed circRNA-mRNA Networks

**Figure 6:**
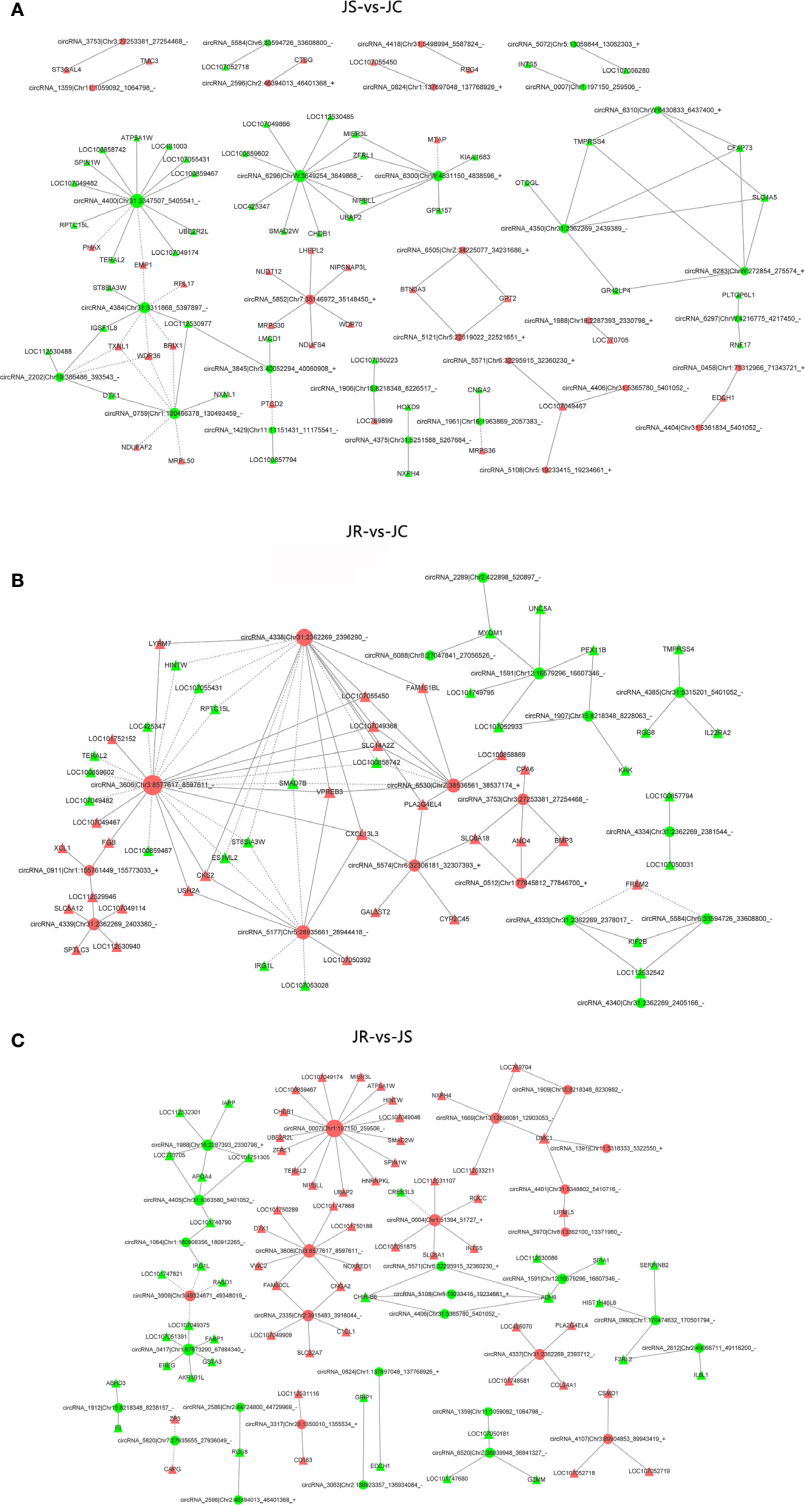
The coexpression network of differentially expressed circRNAs-mRNAs. **(A)** JS *vs* JC group. **(B)** JR *vs* JC group. **(C)** JR *vs* JS group. The red and green represent upregulated and downregulated circRNAs, respectively, while the circles represent the circRNAs, and the triangles represent the mRNAs.

To reveal the function of circRNAs, a coexpression network map between differentially expressed circRNAs and mRNAs was constructed using the *P*-value of hypergeometric distribution. In the top 100 coexpressed circRNA-mRNA network maps, a total of 32 circRNAs and 74 mRNAs were identified in the JS *vs* JC group (**Figure 6A**, **Supplementary Table 4**). Eighteen circRNAs and 55 mRNAs in the JR *vs* JC group were coexpressed in the cecums of *E. tenella*-infected chickens (**Figure 6B**, **Supplementary Table 5**), and 30 circRNAs and 79 mRNAs were coexpressed in the JR *vs* JS group (**Figure 6C**, **Supplementary Table 6**). Among them, in the JS *vs* JC group, circRNA2202 and circRNA0759 were positively correlated with *DTX1* expression. Moreover, circRNA6300 was positively correlated with the expression of *RORC* and *CD101*. CircRNA4338 was coexpressed with *VPREB3* and *CXCL13L3* in the JR *vs* JC group, and circRNA2612 was coexpressed with *IL8L1* and *F2RL2* in the JR *vs* JS group.

## Discussion

Research on the selection of broiler chicken breeds or strains naturally resistant to coccidiosis has been on the way. Johnson and Edgar (1982) reported early that the genetic variability of chickens could affect the resistance and susceptibility to acute cecal coccidiosis (ACC) and confirmed that resistant and susceptible lines of Auburn Strain Leghorn resulted in a sixfold difference in the ACC mortality rate. Later, Quist et al. (1993) showed that coccidia infection in the host cells of the Auburn Strain Leghorn-resistant line was more serious than in the host cells of the resistant line *in vitro*. In addition, *Marek’s* disease and *Necrotizing Enteritis*-resistant and -susceptible strains have been reported (Heidari et al., 2020; Pham et al., 2020). Therefore, the resistance of chickens to pathogens are related to genetic variability. Swaggerty et al. (2011) selected broiler lines with strong innate immunity and showed stronger natural resistance to coccidia, as demonstrated by lower lesion scores and higher weight gain. The coccidial resistance of chickens also depend on the immune capacity of the host. In this study, the chicken groups that are naturally resistant and susceptible to *E. tenella* were selected from different families to explore the molecular mechanism of the differences between susceptible groups and resistant groups.

CircRNAs not only participate in the posttranscriptional regulation of genes functioning as miRNA molecular sponges but also interact with RNA binding proteins (Du et al., 2016). Although the functions of most circRNAs remain largely unexplored, as circRNA functions are being gradually revealed, it is becoming increasingly important to study the circRNA mechanisms in infection and diseases. In previous studies, the expression pattern and regulation of circRNA have been described in porcine endemic diarrhea virus (PEDV) (Chen et al., 2019b), bovine viral diarrhea virus (BVDV) (Li et al., 2019), and crucian carp *Carassius auratus gibelio* (Hu et al., 2019). In poultry, Chen et al. (2019a) found that five differentially expressed circRNAs were upregulated in chicken ammonia poisoning. Liu et al. (2020) reported that 27 differentially expressed circRNAs were involved in the immune response to infectious bursal infection in chickens. Fan et al. (2020) performed transcriptome sequencing on the small intestine tissues of chickens infected with *E. necatrix*. and showed that 13 differentially expressed circRNAs, such as circRNA2673, circRNA3106, and circRNA1579, played an important role in the process of *E. necatrix* infection. In this study, *E. tenella* infection induced the significant alteration of these circRNAs expression. Further, we found that the functional enrichment of these differentially expressed circRNAs were significantly enriched in the adaptive immune response and the B cell receptor signaling pathways. Adaptive immunity is specific and regulates the antigen-specific immune responses to prevent colonization and growth of the pathogen inside the host and the adaptive immune response plays a dominant role in anticoccidial protective immunity (Kim et al., 2019). B cells are the important components of adaptive immune responses in birds (Girard et al., 1997). When coccidia begins to infect the host, B lymphocytes can express antigen-specific surface immunoglobulin molecules that bind to the antigen, and then B cells with the same surface immunoglobulin continue to proliferate and differentiate to participate in host immunity (Lillehoj, 1998). In addition, the natural killer cell-mediated cytotoxicity was the pathway that differentially expressed circRNAs significantly were enriched. Studies have shown that natural killer (NK) cells are involved in defense against invasion of the gut mucosa by coccidia and associated with the innate immunity of coccidial infection (Lillehoj, 1998; Min et al., 2013). Additionally, this study also revealed the potential role of NF-kappa B signaling and the intestinal immune network for IgA production pathways in the process of coccidia infection. These results indicated that these circRNAs are involved in the coccidial infection through affecting chicken innate and adaptive immune response.

To date, a large number of circRNAs have been identified in various species. However, the ways of most circRNAs function are various. Construction of a circRNA-mRNA coexpression network is an effective method to predict circRNA function. In this study, we analyzed the network of circRNA-mRNA coexpression. In the JS *vs* JC group, circRNA2202 and circRNA0759 were positively correlated with the expression of Deltex1 (*DTX1*). DTX1 is an important Notch signaling target and participates in T cell anergy (Matsuno et al., 1998). DTX1 lack enhances T cell activation, enhances autoantibody production, and promotes inflammation (Hsiao et al., 2009). Moreover, DTX1 controls the stability of Tregs at another level *in vivo* by maintaining the protein expression of Foxp3 in Tregs (Hsiao et al., 2015). Therefore, circRNA2202 and circRNA0759 would participate in the immune response of coccidia infection by regulating the *DTXI* gene. In the JR *vs* JC group, circRNA4338 was positively coexpressed with *VPREB3* and *CXCL13L3*. CXCL13 is a member of the chemokine superfamily CXC subtype and is also known as a B cell chemokine 1 or B lymphocyte chemotactic agent (Gunn et al., 1998). VPREB3 belongs to the immunoglobulin (Ig) superfamily and regulates the assembly of the light chain proteins VPREB1 and IGLL1 to form the pre-B-cell receptor (pre-BCR) (Rosnet et al., 2004). The complete pre-BCR plays a vital role in the development of mammalian B lymphocytes (Rodig et al., 2010). Felizola et al. (2015) showed that VPREB3 is expressed in B cell differentiation and mature B lymphocyte subsets. Therefore, circRNA4338 would be involved in the immune response to coccidia infection. In the JR *vs* JS group, circRNA2612 was coexpressed with *IL8L1* and *F2RL2*. Interleukin-8 (IL-8), known as chemokine CX-CL8 (CXCL8), is a proinflammatory factor closely related to the occurrence and development of various inflammatory diseases. IL-8 exerts various biological functions by binding to the receptors CXCR1 and CXCR2, including pro-inflammatory cell chemotaxis, inducing neutrophils to release lysosomal enzymes to eliminate pathogens (Coussens and Werb, 2002), activating immune responses and promoting infected tissue healing (Wang et al., 2020). As a G-protein-coupled protease-activated receptor, F2RL2 plays an important role in the coagulation cascade (Kaufmann and Hollenberg, 2012). Research by Liu et al. (2019) showed that the expression level of *F2RL2* was downregulated during infection with BVDV, suggesting that the complement system may play a key role in BVDV infection. In this study, circRNA2612 would participate in the immune response of the chicken host against *E. tenella* infection by associating with *IL8L1* and *F2RL2*. At present, there are related reports on the functional verification of circRNAs involved in disease and the inflammatory response through regulatory genes (Ma et al., 2018; Zhang et al., 2019). However, few studies have been reported on the functional test of circRNA regulatory genes involved in the immune response to coccidiosis. Therefore, functional verification experiments on the screened circRNAs *in vivo* and *in vitro* are the next research work of our group.

In conclusion, the results of this study demonstrate for the first time that circRNAs are differentially expressed in the cecal tissues of chickens infected with *E. tenella* and may be involved in host responses to *E. tenella* infection in chickens. These findings also provide valuable data for understanding the circRNA complex mechanisms of *E. tenella* resistance and susceptibility in chickens. However, the action mechanisms of circRNAs and mRNAs remain to be clarified.

## Data Availability Statement

The datasets presented in this study can be found in online repositories. The names of the repository/repositories and accession number(s) can be found below: NCBI; PRJNA678759.

## Ethics Statement

The animal study was reviewed and approved by the Animal Welfare Committee of Yangzhou University.

## Author Contributions

HY and GD designed the study and wrote the manuscript. HY, CM, and QW performed the experiments and analyzed the data. HY and WZ performed the transcriptome data and prepared the figures. TZ, GZ, KX, HS, and JW reviewed the manuscript. All authors contributed to the article and approved the submitted version.

## Funding

This research was supported by the Jiangsu Agricultural Industry Technology System (JATS[2020]437), the National Sci-Tech Support Plan (2014BAD13B02), the Priority Academic Program Development of Jiangsu Higher Education Institutions (PAPD), and the China Agriculture Research System (CARS-41-G23).

## Conflict of Interest

HS was employed by Jiangsu Jinghai Poultry Group Co. Ltd.

The remaining authors declare that the research was conducted in the absence of any commercial or financial relationships that could be construed as a potential conflict of interest.
